# Progress of the Medical Sciences

**Published:** 1892-03

**Authors:** 


### Progress of the Medical Sciences.

### MEDICINE.

In these days of therapeutic activity, when nearly every successive issue of every journal chronicles the advent of some new remedy, with a scientific name too unwieldy for ordinary use, and too often a value inversely as the length of its chemical formula, it is a novelty to note the reviving reputation of a long-known simple chemical element.

Oxygen is not now for the first time associated with medicine. The most important of all the elements was not likely to escape the notice of the inquiring scientific physician. A hundred years ago, and therefore soon after the discovery of oxygen by Priestley, Dr. Thomas Beddoes established in the Hotwells, at Bristol, a Pneumatic Institution, the superintendent of which was Mr. (afterwards Sir Humphry) Davy, who made in its laboratory many of his discoveries. Here observations were made upon the therapeutic value of many gases, including oxygen, and much was anticipated from the experiment. In the result, however, Beddoes showed himself "over-sanguine and speculative," and consequently his expectations were not realised. The institution gradually became an ordinary hospital, and in the course of ten years was relinquished by its founder, who is said to have previously branched off in various erratic directions, such as the domiciling of patients with cows and their insertion up to the neck in the excrement of these animals.

Later writers upon therapeutics have, as a rule, touched upon oxygen in a tentative manner suggestive of want of personal acquaintance with it. Scattered references to the gas are met with in medical literature, but it is only of late that its use has been prominently advocated.

Dr. Jacobi[1] speaks of oxygen in the treatment of "membranous croup," and quotes a case in which extension of the membrane took place below the tracheotomy opening, with resulting dyspnœa, cyanosis, and convulsions: whenever a current of the gas was introduced through the cannula, cyanosis and dyspnœa disappeared, to return when the supply was stopped. Delafield[2] recommends its inhalation in acute parenchymatous nephritis. Dr. W. G. Thompson[3] advocates the use of oxygen especially in the dyspnœa of chronic Bright's disease and uræmia, in pneumonia, chronic bronchitis, asthma, and catarrhal bronchitis. Dr. J. Chambers[4] says: "In pneumonic cases presenting symptoms of deficient blood-aëration, the inhalation of oxygen gas has in my hands proved to be a remedy of remarkable power. Under its use the lips recover their redness, the breathing becomes easy, and the toneless heart is strengthened in its action." He has given from half-a-gallon to a gallon of the gas every half-hour for as long as four days and nights with perfect safety, and is convinced that "life has certainly been saved in many cases where it has seemed that death was inevitable." If cardiac weakness is urgent, he also advises the administration of sulphate of strychnine, in doses of $\frac{1}{80}$ of a grain every four or six hours, until a decided change in the pulse is manifest, when the drug should be omitted, to be used again later if the heart tends to fail. Again, Dr. A. N. Blodgett[5] reports a remarkable case of pneumonia successfully treated by the continuous inhalation of oxygen. A lady was attacked with extensive pneumonia, with marked general symptoms. Excessive dyspnœa set in, against which all ordinary measures were useless, and oxygen was therefore administered, with very marked relief; but the dyspnœa returned again and again, though each time staved off by the inhalation, and at last the gas was given continuously. At one time the supply began to fail, and before fresh could

[1] Pepper's *System of Medicine*, 1885, vol. iii., p. 107.
[2] *Op. cit.*, vol. iv., p. 80. [3] *Med. Rec.* July 6, 1889.
[4] *Lancet*, 1890, vol. i., p. 1120.
[5] *Boston Med. & Surg. Journ.*, No. 21, 1890.

be obtained the patient was moribund; oxygen was therefore administered by artificial respiration, and the patient again rallied. The inhalation was then continued for 106 hours without intermission, and at the end of this time the breathing was healthy and natural; and recovery followed without further accident. On an average 200 gallons of oxygen were used in twenty-four hours. The author says that the effect was "almost as pronounced and evident as is that of ligature in hemorrhage."

Special prominence has since been given to this subject by Drs. Lauder Brunton and Prickett.[1] The gas was administered to a patient with double pneumonia, who when seen was "completely unconscious and apparently moribund, his face livid, the skin cold and covered with a clammy sweat, and loud mucous rattles accompanied every respiration." Fifteen ounces of blood were withdrawn by venesection, two doses of $\frac{1}{40}$ of a grain of strychnine were injected subcutaneously with an interval of twenty minutes, and oxygen gas was then administered. In two hours "an extraordinary transformation had taken place: the patient was perfectly conscious, his colour was quite healthy, and he expressed himself as feeling comfortable and well." The inhalation was then discontinued for some hours, when the pulse and respiration again began to fail; and in spite of the continued administration of oxygen, the patient died.

This communication has elicited the experience of other observers.

Mr. T. A. O. Langston[2] gave the gas to a patient with acute bronchitis, following influenza, who was completely insensible, with intense cyanosis, irregular respiration, and a very feeble failing pulse. In two hours the cyanosis was gone, but the patient was still quite insensible; in three hours she was perfectly conscious. When the inhalations were stopped for some hours, insensibility returned, to be again removed when the inhalations were renewed. A few hours later the supply of the gas ran out, and the patient died before more could be obtained. The effect of the oxygen was "simply marvellous," bringing the patient temporarily "out of the jaws of death to life." Dr. W. Carter[2] has for some years used the gas in respiratory troubles. He quotes an instance in which it undoubtedly saved the life of a woman with bronchitis and dilated right ventricle, who was blue and all but pulseless, and evidently about to die. The good effect of the oxygen was soon seen, and the following morning the patient was quite comfortable. Her recovery was uninter-

[1] *Brit. Med. Journ.*, Jan. 23, 1892. [2] *Brit. Med. Journ.*, Jan. 30, 1892.

rupted, and she was discharged from the hospital in due course. In Bright's disease, with threatened uræmia, Dr. Carter follows Jaccoud's plan of administering the gas mixed with air from a portable gasometer, which is wheeled to the patient's bedside. He points out that oxygen is excessively stimulating, and if inhaled suddenly in large amount may cause a tetanic condition of muscle; and also that its sudden expansion on its escape from the cylinder chills the air and the patient, and may cause irritation and cough. He uses an ivory (not glass) mouthpiece, through which the gas flows from the cylinder in a gentle stream that can just be felt on the back of the hand.

Dr. J. C. Thorowgood[1] quotes a case of advanced phthisis in which oxygen relieved oppression and dyspnœa, and caused the return of colour to the lips. The cylinder lay under the bed, and the patient now and again took an inhalation by means of a pipe with a stop-cock.

Dr. Aubrey Blakiston[2] gave the gas in three cases of pneumonia and one of acute bronchitis. All were unusually severe, and all recovered; and he believes that in two of these life was saved by its use. In two out of three cases of bronchial asthma the result was also very satisfactory. In a case of convalescence from acute illness, the patient said that after massage and the inhalation of oxygen he "felt as if he had been a six-mile walk and breathed sea-air."

Dr. A. W. Gilchrist[3] cites a case of influenza with lung complications, in which death was imminent from asphyxia and cardiac failure. Some improvement followed the injection of *two drachms* (!) of liquor strychniæ, repeated after two hours; but as collapse again threatened, strychnine was resumed "in larger doses," and oxygen was administered; with the result that the colour improved and the patient became more sensible and expressed herself as feeling relieved. On the fourth night she again became insensible, with cold extremities and dusky clammy surface. Fourteen leeches were applied, and oxygen was again inhaled: cyanosis was markedly relieved, the pulse improved, and complete consciousness returned. After some hours, however, death occurred, although the gas was still given "at intervals."

Dr. J. L. Porteus[4] of New York has frequently used a mixture of two parts of oxygen and one of nitrogen monoxide in pneumonia, phthisis, asthma, and uræmia. In the cyanosis of pneumonia wonderful effects were produced. In

[1] *Brit. Med. Journ.*, Jan. 30, 1892. [2] *Brit. Med. Journ.*, Feb. 13, 1892.
[3] *Brit. Med. Journ.*, Feb. 6, 1892. [4] *Brit. Med. Journ.*, Feb. 20, 1892.

one case a minute's inhalation reduced the respirations from 50 to 25 per minute, and gave great relief. He advocates its use in the *early stages* of pneumonia. Inhaled for a minute or two every half-hour till the patient is relieved, and then every three or four hours, it lessens the frequency of the respirations, slows the heart's action, subdues restlessness, and induces sleep. In asthma its administration two or three times a day gives excellent results.

My own experience [1] accords with that of the foregoing observers. A patient, aged 66, the subject of marked emphysema and secondary dilatation of heart, was attacked with extensive bronchitis and broncho-pneumonia. When the inhalation of oxygen was begun the pulse was rapidly failing, the surface was very dusky, and the end was fast approaching. The immediate effect was most striking: the pulse improved wonderfully in tone, and the cyanosis completely disappeared. When the inhalations had ceased for a few minutes, however, the pulse again began to fail and the cyanosis to return, to be again removed by the fresh administration of the gas. In about ten hours' time the effect of the inhalations began to be less marked, and the patient gradually sank and died. The influence of the oxygen in temporarily removing cyanosis and stimulating the failing heart was extraordinary, and altogether beyond doubt; and although a fatal issue was not averted, yet it was manifest that life had been prolonged for some hours.

In most of the recent cases the gas has been obtained from Brin's Oxygen Company, 34 Victoria Street, Westminster, whose Bristol agent is Mr. C. H. James, of 30 Broad Street. This company also supplies a simple inhaling apparatus, which is preferable to the tube leading directly from the cylinder to the patient's mouth.

All the observers agree in describing the wonderful influence of oxygen in removing cyanosis and stimulating the heart; but the ultimate result has too often been unfavourable. It is evident, however, that the gas has been given only as a last resource, and when the patient has been even *in articulo mortis;* and its extraordinary temporary power under such disadvantageous circumstances gives ground for hope that, if intelligently handled, it may prove a therapeutic agent of great value. The theory of its administration is sound; increased power of oxidation in the inhaled atmosphere should compensate for diminished oxidising surface. As oxygen forms about one-fifth of atmospheric air, an atmosphere consisting solely of the gas should suffice for the

[1] *Brit. Med. Journ.*, Feb. 6, 1892.

oxidation of the blood if only one-fifth of the normal respiratory area were available. The mathematical accuracy of this calculation it would be difficult to demonstrate; but that it cannot be very wide of the truth, such results as those described by Dr. Lauder Brunton go far to prove.

In conclusion, it may be said that the following are the lessons to be drawn from our present experience of the use of oxygen in acute respiratory troubles:

1. That its administration should not be too long deferred.
2. That in extreme cases it should be given continuously and persistently.

E. Markham Skerritt.

### SURGERY.

Dr. F. N. Otis of New York contributes a paper on "Reflex Irritations and Neuroses caused by Stricture of the Urethra in the Female."[1] The symptoms he describes are such as are frequently observed and described under the head of "irritability of the bladder," and which, when occurring in women, are generally ascribed to abnormal conditions of the urine, uterine or pelvic troubles, or hysteria (all of which can undoubtedly produce the symptoms), but never to what Dr. Otis maintains is a very frequent cause; viz., stricture of the urethra. This condition receives the scantiest notice (if any) in most of the recognised surgical works, and the conclusions arrived at in this paper, if corroborated, will deserve the most careful attention. Two points may be brought into prominence from the observations and cases here published: (1) that the strictures met with are of the "large calibre" kind, and are yet capable of causing the severest reflex symptoms; and (2) that the stricture may be situated at any part of the canal. Dr. Otis finds, from a limited number of exact observations, that the normal calibre of the female urethra varies from 30 to 45 mm. in circumference ($\frac{2}{5}$ to $\frac{3}{5}$ inch in diameter), and that it is of nearly uniform size throughout its length, with the exception of an occasional very slight contraction at the meatus. To determine the existence of any narrowing, the urethrometer or bulbous sounds may be used. In using the former, it should when closed be passed well into the bladder, then fully expanded and withdrawn till it hitches against the "internal meatus." The bulb is then turned down till it will pass through the neck of the bladder, giving the proper calibre of the urethra;

[1] *Med. Rec.*, Jan. 9, 1892.

and as the instrument is gradually drawn out, any lessening of this will be readily detected and accurately gauged. In three cases quoted in the paper, the contraction varied from 8 to 12 mm., and the site from within half an inch of the neck of the bladder to the external meatus.

Divulsion or gradual dilatation may be employed in the treatment of these cases. The former is much less risky in the case of the female than of the male urethra; and Dr. Otis has in several cases carried the distension up to 70 mm.; but he considers gradual dilatation up to the normal calibre the most judicious treatment as a general rule. The tendency to recontraction of the stricture seems to be very great, but the occasional passage of an instrument will keep up the good effect, and this many patients can learn to do for themselves.

In reference to the causation of these strictures, Dr. Otis insists that they can arise, quite independently of any venereal origin or noticeable urethritis, from the passage of irritating urine in lithiasis. This he maintains is true in both sexes.

Van de Warker [1] supports Dr. Otis's opinion as to the frequency of stricture in women, and advises gradual dilatation.

Dr. A. T. Cabot of Boston supplies a valuable paper [2] on the subject of the surgical treatment of a calculus impacted in the ureter, based on a consideration of the anatomy of the tube and on experimental dissections. He starts with the assumption that extraperitoneal ureterotomy is the operation of choice, as the ureter is so thin-walled that its safe closure intraperitoneally is very uncertain. The anatomical relations on which most stress is laid are:

1st. That the ureter is adherent to the overlying peritoneum, so that it is separated with it when the latter is stripped up.

2nd. That its position with regard to the line of firm adherence of the peritoneum to the spine is very constant, being just outside it, but a little further out on the right side owing to the interposition of the vena cava, with which it is in close apposition.

3rd. That the converging lines of the ureters through the pelvis lie pretty closely over the lateral edges of the sacrum.

4th. That, in the female, the ureter for the last two, or sometimes three, inches runs in the broad ligament in close relation to the upper part of the vault of the vagina.

[1] *Journ. Amer. Med. Assoc.*, Oct. 4, 1890.

[2] *Amer. Journ. Med. Sci.*, Jan., 1892.

On this basis he proceeds to formulate various operative measures for exposing the ureter in any part of its course. In its abdominal course he adopts the incision planned by Israel—a line drawn from the outer edge of the erector spinæ, a finger's-breadth below the last rib, parallel with it to its tip, then turning downwards and inwards to a point about two inches above and internal to the anterior superior iliac spine, whence it is continued in a curved direction to a point about an inch above and external to the middle of Poupart's ligament, and then inwards to the external border of the rectus muscle. According to the position of the calculus, the incision is to be made in the posterior, middle, or anterior third of this line. This excludes the pelvic part of the ureter —the last three or four inches—in adults at any rate, the part, unfortunately, where calculi most often lodge. Sometimes the stone projects through the mouth of the ureter into the bladder, or lies in the vesical part of the ureter merely covered by vesical mucous membrane. In either of these cases it may be removed through the bladder; but if it be impacted just outside the bladder, the danger of urinary infiltration is too great to allow of its removal in this way. For such cases Dr. Cabot has devised the following operation: An incision is made along the border of the sacrum, stopping just below the point of the coccyx, the sacro-iliac ligaments are divided, and the coccyx and lower part of that side of the sacrum are removed. This gives free access to the ureter, and its relation to the edge of the sacrum will serve as a guide to its position, although the presence of a stone will probably remove all difficulty on this head. The peritoneum is very thin, and so would be very liable to be torn if the search were protracted. The rectum had better be defined by introducing a large sound, which can at the same time be used to draw it to one side.

In the female this part of the ureter can be reached by a much less formidable operation. An incision made outwards and backwards through the upper part of the vault of the vagina will reach it between the layers of the broad ligament. The finger is pushed up into the ligament separating the tissues, till the stone is reached. Dr. Cabot relates a case in which he successfully practised this method. The stone, which was felt as a hard, very sensitive, mass close to the cervix, was easily reached by the vaginal incision. Its extraction was not so easily accomplished, as the presenting end of the calculus broke off in the forceps, and the remaining portion had to be dislodged by a blunt hook passed into the ureter above it. She made a good recovery, with the exception of a small fistulous opening, which persisted when the patient was last heard of, four months after the operation. No urine came through the fistula, showing that the corresponding kidney had been completely destroyed.

Dr. Cabot next discusses the question of localisation when the symptoms of calculous impaction are present. A stone impacted in the lower few inches of the ureter can generally be felt by a rectal or vaginal examination; but in the higher part of its course palpation is very difficult and uncertain. A spot of constant tenderness in the line of the ureter has sometimes indicated the place of impaction. But if, as must often happen, no indications are present to locate the stone, an exploratory abdominal incision should be made, and the whole length of the ureter examined. Dr. Cabot refers to two cases where this was done by Hall[1] and Arbuthnot Lane[2] with most satisfactory results: the calculus was easily found, and then removed extraperitoneally by another incision in the lumbar region. Lane's case is especially instructive, inasmuch as the pelvis of the kidney and the upper part of the ureter had been previously explored through a lumbar incision, and the lower part through the rectum and vagina, and at the subsequent abdominal exploration the stone was found in that part of the ureter which could not be reached from the lumbar wound or from below. The calculus was manipulated upwards to the crest of the ilium where, it could be easily reached from the loin—a manœuvre worth bearing in mind. Mr. Lane closed the ureteral incision successfully with silk sutures; but Dr. Cabot objects to this method, that it is very difficult to carry out securely without allowing any of the sutures to pierce the mucous membrane, in which case they might serve as nuclei for fresh calculous formation. The incision seems to heal readily and soundly when left alone.

Iodoform has long enjoyed a reputation in the treatment of tubercular lesions, and its utility is the subject of an elaborate paper by Dr. Senn in the *Annals of Surgery*.[3] After summarising the experiments of numerous observers in this direction with various antiseptic substances, he discusses the results of laboratory and clinical experience in the case of iodoform. Experiments by Troje and Tangl are referred to as showing that iodoform dusted over culture media markedly diminishes the virulence of tubercle bacilli; and others by

[1] *Med. Rec.*, Oct. 18, 1890. [2] *Lancet*, Nov. 8, 1890.

[3] Jan., 1892: "Treatment of Tuberculosis of Bones and Joints by Parenchymatous and Intra-articular Injections."

Gosselin on rabbits and guinea-pigs, in which the prolonged use of iodoform injections seemed undoubtedly to retard, and in some cases altogether prevent, the extension of previously inoculated tuberculosis.

The clinical experience in its favour is however the one to which Dr. Senn appeals as the most uniform as well as convincing, and he quotes the testimony of Billroth, Krause, Bruns, Trendelenburg, and many more in its favour. The method advocated is that of injecting iodoform, held in suspension, either interstitially into solid tubercular foci, or into the cavity of a tubercular joint or abscess. The injection can be made either through a cannula or by means of an ordinary injecting syringe, the puncture being sealed with a piece of aseptic wool soaked in iodoform collodium. The precautions that require emphasising are:

1st. A most careful disinfection of the skin and injecting instrument.

2nd. The use always of a freshly-prepared and sterilised emulsion of iodoform in glycerine or olive oil (10 per cent.).

3rd. A sufficient interval between the injections.

The initial dose varies according to the age, and the size of the tubercular focus; but 3 to 8 drachms is an average dose. This should not be repeated oftener than every eight or fourteen days. Employed in this way, the injections are followed by no dangerous complications. An ethereal solution must be avoided, as gangrene of the overlying tissues and iodoform intoxication have frequently followed its use. In the case of tubercular joints containing fluid, this should be first withdrawn through a cannula and the joint irrigated with a 3—5 per cent. solution of boracic acid till it returns quite clear, and then injected, taking special care to exclude air. If the fluid is too thick to run through a large cannula, the joint should be freely incised, scraped, and rubbed out with iodoform gauze, the wound sutured, and the injection then proceeded with as before. The same rules apply to abscesses. If, on the other hand, the joint contains no fluid, besides throwing the emulsion into the joint-cavity, the thickened synovial (and, if necessary, osseous) tissue should be injected; and it is best to deposit the iodoform in several places, which can be done by pushing the needle in different directions without removing it completely. The injection should be made at different points at each repetition of the process. Uniform diffusion of the injected fluid can be secured by kneading the parts, and by passive movements of joints. Dr. Senn does not consider immobilisation of the affected joint necessary during this treatment, unless active movements are very painful. He makes an exception in the case of spinal caries and severe hip-disease; but general surgical experience would suggest its co-operation in all cases.

Bruns and Nauwerk have excised and examined pieces of tubercular tissue at various periods after the injection of iodoform, and as a result of their examination they find that the bacilli disappear, the miliary tubercles become softened by infiltration with round cells and serous fluid, and later that the tubercles disappear by fatty degeneration and liquefaction. Concurrently with these changes a process of repair goes on in the adjacent tissues, forming a line of demarcation in the shape of a wall of healthy granulation tissue, which partly consumes and partly casts off the degenerated tubercular tissue. This being its mode of action, it cannot reasonably be expected to effect a cure where the primary focus contains large masses of cheesy material or a large sequestrum, which must be removed before repair can take place; but the injections do no harm, if they do not actually improve the conditions for surgical measures as Dr. Senn believes.

The paper concludes with a brief report of ten cases treated by the author since April, 1891, with seven good results. In no case were any bad effects produced by the injections. These were always followed by some swelling for a day or two, which afterwards subsided. Improvement was announced by a change in the character of the effusion—the solid portions disappearing, and the fluid becoming viscid and mucus-like shortly before it finally ceased.

W. M. Barclay.

### OPHTHALMOLOGY.

A valuable paper by J. B. Lawford and Treacher Collins appears in the Royal London Ophthalmic Hospital *Reports*, December, 1891. The authors tabulate 103 cases of undoubted sarcoma of the uveal tract, their main object being to obtain evidence on the question of prognosis in this affection. In 95 cases, simple enucleation was performed; in 16, secondary or more radical operations. Of the total number of cases, 79 have been traced—half of these were dead. Of those living—in one-half, three or more years have elapsed since the enucleation was performed; in the other half, the operation was less than three years ago, so that a permanent recovery of one-quarter of the whole number may be assumed. Of those which are known to be dead, one-half died during the first two years after the operation, and three-fourths during the first four years. No pains were spared in seeking for the real cause of death: besides help from several medical men, the death register at Somerset House was consulted in many cases. In at least one-third of the cases traced, death was due to metastatic growth, very frequently in the liver; and in another group the cause of death was assumedly metastasis, although the death returns are not definite upon this point.

Dr. Hill Griffith [1] found that in 23 cases of enucleation for choroidal sarcoma, fourteen (60 per cent.) were well at periods varying from three to ten years; six died of sarcoma of the liver; the other three died, but less certainly from extension of the disease. Local recurrence in the orbit took place in two. He is of opinion that metastasis is not more probable where enucleation has been postponed to a late stage of the eye disease, than where early removal of the eye has been practised. He referred to several cases which had remained well years after enucleation, even though this was done after the growth had become extra-ocular, and in one case, where the tumour had existed as long as seventeen years. Most surgeons would, however, consider it of extreme importance to remove the eye at the earliest possible period after the diagnosis has been made; but Fuchs also found that metastasis was as likely to occur if the operation is performed early as late in the eye disease, while the earlier it is done the less probable is local recurrence. Statistics indicate that local recurrence after operation occurs in from 10 to 25 per cent. of the cases; and in a large majority of these, within about six months of the enucleation.

Sarcoma of the iris must be very rare. Lawford and Collins only mention, in their list, one case. Martin [2] mentions one out of 43 cases; but Fuchs[3] gives 16 out of 259 cases. The choroid behind the ciliary body is almost always the site of these tumours. The ciliary body according to the three authors quoted was the primary seat of the new growth in 6 out of 103, 22 out of 259, and 4 out of 43 cases, respectively. The record of Fuchs, that of 22 cases under his own observation at least 11 died of metastatic growths, *i.e.* one-half, may in all probability be nearer the real average than the 18½ per cent. deaths by metastasis deduced by that author, from his collected list of 259 cases.

Hirschberg,[4] in 1882, gave details of 13 cases of enucleation for choroidal sarcoma. In five cases less than two years had elapsed since the enucleation—not sufficient time for draw-

[1] *Ophth. Rev.*, 1891, p. 353. [2] *Prognostik der Uveal Sarcom.* Halle, 1885.
[3] *Das Sarcom des Uvealtractus.* Wien, 1882. [4] *Arch. f. path. Anat.*

ing conclusions. Of the other eight, six had died ; one from local recurrence six years after operation, five from metastatic formations, without local recurrence, and all five within two years of the removal of the eyeball. Metastasis probably very seldom occurs later than five years from the affection of the eye ; yet Lawford and Collins show that in several cases this fatal complication has attacked the patient at a much later period.

In Lawford and Collins's 103 cases of sarcoma of the uveal tract, 27 occurred between the ages of 40 and 50; 84 cases, between 30 and 70 years; 10 cases before 30, the youngest of all being 15 years old; and 9 cases after 70.

Sarcoma of the uveal tract, is probably about twice as common as glioma of the retina—the latter is usually found in infants. Indeed, Hirschberg believes it is always *ab initio* congenital—it is exceedingly rare at so late an age as fifteen years. Sarcoma in both eyes is, probably, much more rare than binocular glioma.

Mitvalsky[1] (Prague) writes on metastatic carcinoma of the eyeball. He mentions altogether 11 cases. The seat of the primary neoplasm in all save one was in the mammary gland, and one of these patients was a male, in the remaining case the primary growth appeared in the pleura. The ocular metastases were always in the choroid, the first development probably occuring within the lumen of the smaller vessels in the choriocapillaries. Histologically the metastatic growth corresponded in type with the primary one. In at least four of the cases, both eyes were affected; one shortly after the other. The time that elapses between the primary and secondary growths is quite uncertain; but, as in other organs of the body, the metastasis is an indication of the approaching death of the patient.

Dr. Elschning [2] now reports a carcinoma of the choroid which appeared about nine months after removal of the breast and axillary glands. The earlier symptoms resembled detachment of the retina, which continued to increase ; then came increased tension of the eyeball, and afterwards severe glaucoma. The autopsy showed carcinomatous tumours in the brain, lungs, and liver. From careful microscopical examinations of his own case and a study of others, Elschning considers that the metastases are caused by carcinomatous emboli in the choroidal vessels; the epithelial masses develop at the point of invasion within the blood-vessels, they spread first within the vessels, then break through the wall and spread atypically, and cause hyperplastic tissue changes

[1] *Arch. Ophth.*, vol. xx., 2. [2] *Arch. Ophth.*, vol. xx., 3.

in the stroma of the choroid. Usually the tumour within the eyeball is flat, slowly growing, and not easily recognised either by the ophthalmoscope or by its effect on the vision of the patient until the retina becomes detached. In two or three cases on the other hand, the growth has been elevated, and vision has been more rapidly destroyed. Elschning then goes on to consider metastatic sarcoma of the choroid, which he thinks, may be in reality rather more common than authors believe. He says some cases can be best explained by considering the tumour in the eye as a metastatic manifestation of a general sarcomatous infection, whose starting point is to be sought for outside the eye. Though it may no longer be held that melanotic sarcoma is found primarily *only* in the uveal tract, and that thus it is just possible that a melanotic growth found in the choroid may be secondary to one existing elsewhere; still, it is certain that clinically sarcoma of the choroid as a secondary growth must be very rare. A few cases have been quoted by Brömser [1] and by Pflüger.[2]

Gayet [3] has described an adenoma of the choroid secondary to adeno-carcinoma of the stomach.

The differential diagnosis between subretinal tumour and simple retinal detachment, often presents considerable difficulty. The method suggested by Lange of intra-ocular examination through the dilated pupil, with help of strong oblique illumination upon the outer surface of the eyeball through sclera and conjunctiva, shows not only clearly the detached retina, but to a certain extent the kind of substance that lies beneath it.

In acromegaly the pituitary gland has been often found to be hypertrophied, and Dr. Marie [4] considers this one of the essential elements in the pathology of the disease.[5] In a very careful examination of the skeleton in one of these cases, Dr. Alexis Thomson [6] drew attention to the very deeply distended pituitary fossa, due to pressure of the hypertrophied pituitary body.

M. Pinel Maisonneuve [7] has recently called attention to the eyes in a typical case of acromegaly, the symptoms of which might all have been due to compression of the optic

[1] *Arch. f. Ophth.*, vol. xxxi. [2] *Arch. f. Augenh.*, vol. xiv.

[3] *Arch. d'ophth.*, May, 1889. [4] *Rev. de med.*, April, 1886.

[5] *Acromegaly.* Syd. Soc. ed., 1891, p. 61.

[6] *J. Anat. & Physiol.*, 1890. [7] *Progrès méd.*, May, 1891.

chiasma and optic tracts and the back of the orbits by an enlarged pituitary body. There were present exophthalmos, imperfect movement of the eyeballs with want of association between the eye and the lid movements, sluggish pupillary action to light, and congestion of the retinal veins.

Optic atrophy and hemianopsia without interference with the globe or its movements, in a patient with acromegaly, were quoted by Meyer; and Dr. Ruttle,[1] in reporting a case, says: "At the time of the severe explosion, her extremities rapidly enlarged, so much so that her left ring-finger burst her wedding-ring, a moderately stout one. She also began to experience failure of sight at this time, which has gone on to total blindness in the right eye from atrophy of the optic nerve. In the left eye the temporal half of the field of vision is gone, and the activity of the upper limit at the nasal side is slightly enroached on; the hemianopsia is very clearly defined . . . the pupils are always somewhat dilated, the optic axes are divergent." In Saundby's case, no brain tumour could be found, and in one reported by Waldo [2] the pituitary body was healthy; cavities were found on both sides of the cerebrum and of the cerebellum, but no abnormal symptoms or appearances could be detected in connection with the eyes.

Dr. Hadden's case, [3] one of the first published, had optic nerve atrophy of the right eye, with very tortuous veins.

Dr. König[4] draws attention to the excessive secretion from the conjunctiva and from the lacrymal glands which occurs in some cases of locomotor ataxy. He and Féré, who has also noticed a similar condition, suggest the term Lacryorrhœa to express this hypersecretion, which seems to depend on nervous influence, either paralysis of the cervical sympathetic, or irritation of the trigeminus. In one of König's cases the amount of the secretion varied according to the lightning pains, increasing with them in intensity and the converse; the same case presented marked paralysis of both third nerves.

Dufour [5] quotes three cases from Landolt's clinic of bilateral paralysis of the sixth nerves. In two of them the pupils were small, and reacted to accommodation much more freely than to light. Prior temporary attacks of diplopia had been

[1] *Brit. Med. Journ.*, 1891., vol. i., p. 697. [2] *Brit. Med. Journ.*, March, 1890. [3] *Brit. Med. Journ.* 1888, vol. i., p. 855. [4] *Progrès méd.*, Oct., 1891. [5] *Progrès méd.*, Sept., 1891.

present in two of the cases. The patients found difficulty in standing with their eyes closed, and one had total absence of knee-jerk. None of them were distinctly ataxic, but the symptoms seemed to be obviously due to chronic cerebro-spinal mischief, probably an early stage of locomotor ataxy. Macinlay, Doyne and Donaldson, in *Transactions of Ophthalmological Society*, have reported cases of bilateral paresis of the external rectus, but none of these showed any association with locomotor ataxy, and I have recently had a case in which neither eye could be everted beyond the middle line. The patient, a healthy young woman of 25, had recently suffered from "La Grippe." The defect of the eyes was noticed during the illness, a week after its commencement; there was no other defect of muscle power or reflex, either ocular or general. The right eye was the first affected, and is improving; the left external rectus is also gaining slowly in power. The prognosis in this case seems to be absolutely favourable.

Dr. Ferguson[1] recommends strophanthus in the treatment of exophthalmic goitre, from an experience of its use extending over eight cases, in which he has found it more beneficial than any other drug or remedial measure. He gives a dose of the tincture at each meal, commencing with 8 drops and increasing it up to 15 or 25 drops.

Trousseau[2] reports highly of the treatment of chronic types of conjunctivitis by petroleum, which produces less reaction than copper sulphate or silver nitrate. The application is not painful, and the drug is well borne by the inflamed cornea. "Brossage" with a hard tooth-brush was strongly recommended by Osio and others at the recent Spanish Medical Congress, for chronic granulations of the conjunctiva, and such a method might be used in order to rub the petroleum well into the tissues.

Stevens suggests, for the detection of heterophoria, a metal or rubber disk the size of the ordinary trial lens in which a central hole of 3 mm. contains a strong lens about +13D. Such a lens converts the image of a candle flame into a disk of light, the position of which compared with that of the candle flame as seen normally with the second eye, indicates the direction of the faulty tendency. E. B. Myrowitz, 295 Fourth Avenue, New York, makes the disk.

[1] *Am. J. Ophth.* [2] *Centralbl. f. d. ges. Therap.*, Oct., 1891.

C. S. Bull[1] reports five cases of treatment of detached retina by Schöler's method, injection of three drops of tincture of iodine into the vitreous; no good effect occurred in any of the cases, and in more than one severe intraocular inflammation resulted, although the injection was very carefully done and with strict antisepsis.

For immature cataract Fulton[2] advocates extraction without iridectomy, and irrigation of the capsule with tepid 2 per cent. solution of boracic acid, just as soon as the patient is unable to follow his avocation; he leaves bandages off the eye as early as possible, the second or third day. In his opinion, any of the methods suggested for artificial maturation are attended by danger, and are only rarely successful.

F. R. Cross.

### OTOLOGY.

*The Annals of Ophthalmology and Otology* is the title of a new quarterly journal edited by Dr. James P. Parker, and published in Kansas city. The first number, dated January, 1892, contains 74 octavo pages, devoted mostly to original communications. The *Journal of Laryngology and Rhinology* is now called the *Journal of Laryngology, Rhinology, and Otology*, Dr. Dundas Grant being the editor. In addition to these, there is the American *Archives of Otology;* so that the English-speaking race need not remain long in the dark with reference to modern progress in otology.

Dr. Polo of Nantes records a case[3] of trephining the cranium for cerebral abscess following ear disease, which was, to a certain extent, successful, although the patient did not recover.

The patient was a boy aged six, who developed an acute inflammation of the middle ear while convalescing from measles. This was accompanied by great pains in the affected (left) side of the head, not relieved when purulent discharge from the meatus succeeded. When seen by Dr. Polo (a week after the illness commenced) the patient was pale, with slow pulse, responding slightly to questions, and taking little notice of his surroundings. He slept badly, vomited at intervals, passed his urine into the bed, and constantly complained of the left side of his head, where he

[1] *Med. Rec.*, Jan. 16, 1892. [2] *Am. J. Ophth.*, 1891, p. 165.
[3] *Revue de Laryngologie, d'Otologie et de Rhinclogie*, Jan. 15, 1892.

frequently placed his hand. There was internal strabismus of left eye. A perforation was seen in the postero-inferior segment of the left drum-membrane, and the meatus contained pus. The mastoid process was well developed, but neither red nor swollen. Pain was produced by pressure in the mastoid region or above the ear. Pressure became less painful towards the left temple. There was no paralysis of the limbs, and sensation was everywhere perfect.

It was evidently meningitis or a cerebral abscess. The slight rise of temperature, the localisation of the pain, as well as the larger proportion of cases of brain abscess, made Dr. Polo incline to the latter hypothesis; but he decided at first to trephine the mastoid antrum. Nothing was found here, and no relief was afforded; so the next morning he trephined the cranium over the temporo-sphenoidal lobe. A crucial incision was made 4½ centimètres above the auditory meatus, and a large trephine applied. The brain bulged strongly into the trephine hole, and the dura mater having been cut across, the cannula of an aspirator was thrust in, to the depth of three centimètres. Nothing having escaped, a second thrust was made in a backward direction, also without result. At last the instrument was thrust perpendicularly inwards to a depth of four centimètres, with an immediate escape of two teaspoonfuls of moderately thick yellow pus. The aspirator was then applied to the cannula, and some thin clear fluid came away, which resembled cerebro-spinal fluid, evidently coming from the lateral ventricle. An incision was then made by the side of the cannula, and a drainage-tube inserted. Very little amelioration of the symptoms resulted, and the patient died the next day.

Dr. J. Ward Cousins has been exercising his inventive genius again, and has lately described and figured [1] an aural speculum and head-rest for facilitating the examination of the auditory meatus and drum membrane. The head-rest consists of a strong wooden stem, with a handle fixed near the upper end for the patient to hold, and a support for the head, well padded and covered with leather. The peculiarity of the speculum is, that it has a mirror and lens attached to it. In examining the ear the position of the mirror is regulated so that the pencil of light falls upon the bottom of the meatus, and this is then looked at through the lens. I have no doubt that, in the hands of Dr. Ward Cousins or any other surgeon who habitually used it, this instrument

[1] *Lancet*, Jan. 16, 1892.

would afford a good view of the parts looked at; but all such complicated apparatus should be avoided as far as possible, especially in teaching, and a plain forehead mirror and a conical speculum are quite sufficient for all practical purposes.

It is now generally accepted that the auditory centre lies in the first or in the two upper temporal convolutions; but Dr. Charles K. Mills, neurologist to the Philadelphia Hospital, records a case [1] which seems to show that this centre, as well as that of speech, lies in the left half of the brain.

The patient, a woman aged 31, right-handed, had an apoplectic attack, which left her word-deaf but not paralysed. Prior to this attack her hearing had been good; but after it she could not, by hearing, understand anything that was said to her, although she could hear some other loud sounds. Six years afterwards she had another and more severe apoplexy, after which her deafness increased for sounds as well as words, until it was almost total. This seizure left her also with partial left hemiplegia, chiefly affecting the arm. She died nine years after the second attack. At the *post mortem* examination the first temporal convolution of the left hemisphere was remarkably small, narrow, and smooth, except at its anterior extremity. Its posterior two-thirds or three-fourths were shrunken to a thin strip. At a point about the middle of the gyrus, the convolution had so disappeared as to leave only a notch and shred of tissue. At a position corresponding to the posterior fourth of the second temporal convolution and the parallel fissure, the brain presented a marked depression or cavity, at the bottom of which, when the specimen was in the fresh state, was a small mass of yellow, shrivelled, puckered tissue, evidently the remains of an old embolic softening. In the *right* hemisphere was an old and very extensive hemorrhagic cyst which completely destroyed the first, and almost completely the second, temporal gyrus, as well as the island of Reil and part of the ganglia and capsules.

From a study of this case Dr. Mills concludes that the centre for word-hearing is situated in the hinder thirds of the first and second temporal convolutions, just in front of the posterior extremity of the fissure of Sylvius; also that the auditory centres have their highest development in the left hemisphere; but destruction of the auditory areas of the two upper temporal convolutions of both hemispheres is necessary to complete brain deafness.

[1] *Brain*, Winter Number, 1891.

Mr. Arbuthnot Lane calls attention [1] to the great importance of examining the mastoid antrum in certain cases of chronic purulent otitis media. In many of these the foul and caseating inflammatory products collect in the antrum, and produce much irritation by their presence, in consequence of which the size of the cavity is increased by the absorption of its bony walls. Finding this condition to be a great obstacle to cure by simple means, he proceeds to cure the disease by gouging away the whole of the mastoid process and subjacent antrum, allowing the cavity to granulate up from the bottom. At the same time, the opening between the antrum and the tympanum is enlarged, so as to freely drain the tympanum through the aperture, and allow currents of antiseptic fluid to be syringed through. A very successful case is described and reference made to other cases of the same kind lately published by the same surgeon. [2]

W. H. HARSANT.

### Reviews of Books.

[1] *Guy's Hospital Gazette*, Feb. 6, 1892. [2] *Lancet*, Sept. 26, 1891.

